# Effects of Macleaya Cordata Extract on LPS-Induced Intestinal Inflammation and Diarrhea via Modulation of Gut Microbiota

**DOI:** 10.3390/ani16121922

**Published:** 2026-06-22

**Authors:** Jialu Huang, Yue Su, Kaijun Wang, Peng Huang, Wangping Zhou, Jianguo Zeng

**Affiliations:** 1 Hunan Institute of Animal and Veterinary Science, Changsha 410131, China; huangjialu18@hunaas.cn; 2 Chinese Medicinal Materials Breeding Innovation Center of Yuelushan Laboratory, Changsha 410128, China; kj-wang@foxmail.com (K.W.); huangpeng@hunau.edu.cn (P.H.); 3 Key Laboratory of Systems Health Science of Zhejiang Province, School of Life Science, Hangzhou Institute for Advanced Study, University of Chinese Academy of Sciences, Hangzhou 310024, China; suyue@ucas.ac.cn; 4 College of Veterinary Medicine, Hunan Agricultural University, Changsha 410128, China; 5 Hunan Provincial Key Laboratory of the Traditional Chinese Medicine Agricultural Biogenomics, Changsha Medical University, Changsha 410219, China

**Keywords:** *Macleaya cordata* extract, lipopolysaccharide, gut microbiota, diarrhea, acetic acid

## Abstract

Diarrhea significantly impacts livestock and poultry health, causing growth delays and higher mortality rates. Macleaya cordata extract (MCE) demonstrates strong antioxidant, antibacterial, and anti-inflammatory properties, indicating its potential as a therapeutic agent for diarrhea. This research investigated whether MCE alleviates lipopolysaccharide (LPS)-induced diarrhea in mice through modulation of the gut microbiota. Here, changes in short-chain fatty acids (SCFAs) and gut bacterial structure were analyzed using gas chromatography–mass spectrometry (GC-MS) and 16S rRNA gene sequencing, respectively. The effects of MCE administration (40 mg/kg) on intestinal injury and inflammatory responses were assessed in mice induced with LPS. These results show that MCE-treated mice exhibited significantly lower diarrhea indices, attenuated duodenal villus shortening, and decreased crypt depth compared with LPS-induced mice. MCE treatment substantially reduced the mRNA expression of IL-6, IL-1β and NF-κB in the duodenum, as well as the serum levels of TNF-α and IL-8. Furthermore, MCE significantly increased SCFA levels, particularly acetic acid, and reshaped the gut microbiota composition by increasing the abundance of Lachnospiraceae. Given the close interaction between gut microbiota, microbial metabolites, and host inflammatory responses, these microbial and metabolic alterations are closely associated with the attenuation of intestinal and systemic inflammation. In conclusion, the protective effects of MCE against LPS-induced diarrhea in mice are closely associated with the modulation of gut microbiota structure, suppression of inflammatory responses, and enhancement of acetic acid production. This study provides a mechanistic basis for MCE as a natural alternative to antibiotics for treating inflammatory diarrhea in livestock and poultry.

## Simple Summary

Macleaya cordata extract (MCE) effectively reduces LPS-induced diarrhea and repairs intestinal damage in mice by lowering inflammation and balancing gut microbiota composition. Findings show that MCE treatment increases beneficial acetic acid production and restores healthy bacterial populations, such as Lachnospiraceae, providing a natural alternative to antibiotics for treating livestock diarrhea.

## 1. Introduction

Diarrhea results from disruption of the intestinal environment and is often accompanied by dehydration and impaired nutrient absorption [1]. Pathogen-induced diarrhea damages intestinal epithelial cells, which are essential for the absorption of nutrients [2], and leads to compromised mucosal barriers, electrolyte imbalance, altered enzyme activities, and impaired digestion and absorption [3]. During severe bacterial infections, Gram-negative bacteria release lipopolysaccharides (LPSs) because of cell wall disruption [4]. LPS is a potent activator of the innate immune system and is recognized as a key risk factor for intestinal inflammation [5]. LPS-induced acute diarrhea is associated with vascular injury [6] and activation of the NOS II-GC-S autocrine signaling pathway [7]. Excessive LPS exposure promotes inflammatory cytokine production, disrupts intestinal epithelial tight junctions, and ultimately leads to clinical manifestations such as diarrhea and intestinal bleeding [8,9].

Antibiotics have historically been employed in the management of diarrhea; however, their extensive use has raised serious concerns regarding antimicrobial resistance and environmental safety. As a result, the European Union implemented a prohibition on the use of antibiotic growth promoters in animal feed in 2006, which was subsequently mirrored by a comparable ban in China in 2020 [10]. In this context, bioactive compounds derived from plants have garnered significant attention as safe and effective alternatives [11]. Natural medicinal plants contain a wide range of active constituents that can enhance digestive secretion, maintain intestinal barrier integrity, and exert antibacterial, anti-inflammatory, and antioxidant effects [12,13,14,15]. Coptidis rhizome is a traditional Chinese medicinal herb whose major active component, berberine, has long been used for the treatment of diarrhea associated with intestinal infections [16]. As a benzylisoquinoline alkaloid, berberine exhibits multiple pharmacological activities, including suppression of inflammation, regulation of gastrointestinal motility, improvement of impaired intestinal function, and reduction of intestinal secretion and exudation [17].

*Macleaya cordata*, a member of the papaveraceae family, has been used as a traditional antibacterial medicinal plant since the Tang dynasty. This plant is rich in benzylisoquinoline alkaloids, including sanguinarine, chelerythrine, protopine, and allocryptopine [18]. *Macleaya cordata* extract (MCE, sanguinarine + chelerythrine ≥ 60%), which primarily contains sanguinarine and chelerythrine, has been reported to exhibit strong antibacterial and anti-inflammatory activities [19]. Previous studies have demonstrated that MCE enhances antioxidant capacity, suppresses infection, and promotes intestinal development and growth performance [16,20,21,22]. Advances in sequencing enable deeper studies on how diet affects animal gut microbiota [23,24,25,26]. Metagenomic analyses further suggested that MCE can modulate gut microbiota composition, increase beneficial bacteria such as *Lactobacillus*, and enhance SCFA synthesis [19]. Although low-dose MCE has been studied as a growth promoter, its precise metabolic and mechanistic roles in regulating diarrhea symptoms, gut microbiota, and microbial metabolites during acute intestinal injury remain completely unknown.

Changes in gut microbiota composition are strongly linked to diarrhea and intestinal inflammation. Therefore, elucidating microbiota and metabolite changes induced by MCE may provide mechanistic insights into its antidiarrheal effects. In the present study, an LPS-induced intestinal injury model was established to evaluate whether MCE could alleviate inflammatory diarrhea by modulating gut microbiota structure and SCFA production. Moreover, both MCE and berberine are rich in benzylisoquinoline alkaloids and exhibit antidiarrheal effects, but their underlying mechanisms remain poorly compared. The effects of MCE were compared with those of berberine to further clarify its therapeutic potential and underlying mechanisms.

## 2. Materials and Methods

### 2.1. Chemicals and Reagents

MCE (sanguinarine + chelerythrine ≥ 60%) was obtained from Hunan Micolta Bioresource Co., Ltd., with batch number 112025-60-2 (Changsha, China). Berberine hydrochloride was purchased from Northeast Pharmaceutical Group Shenyang No. 1 Pharmaceutical Co., Ltd. (Shenyang, China). Lipopolysaccharides (LPSs) (*Escherichia coli* 055: B5) were provided by Beijing Solarbio Science & Technology Co., Ltd. (Beijing, China). Other chemicals, solvents and reagents were all of analytical grade.

### 2.2. Animals and Experimental Design

**Figure 1:**
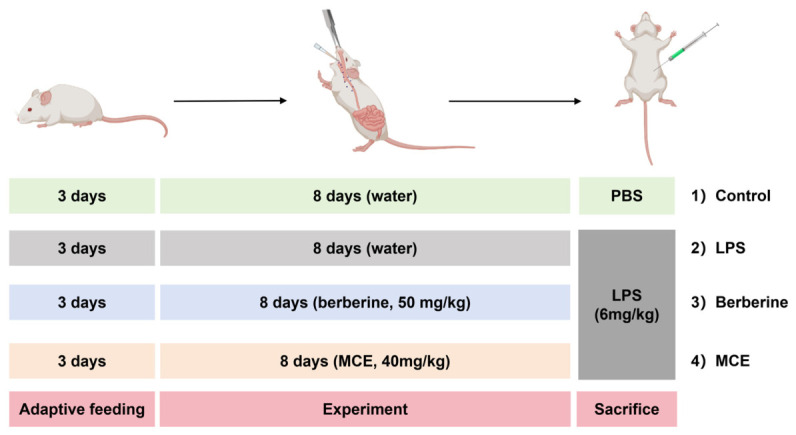
Experimental design. MCE = *Macleaya cordata* extract, LPS = lipopolysaccharide. The berberine group was given berberine (50 mg/kg) and the MCE group was given MCE (40 mg/kg) by oral gavage once a day for 8 days (days 1–8), and the control group and LPS group were administered water every day. Except for the control group, which was administered PBS intraperitoneally, the remaining groups were treated with LPS (6 mg/kg) intraperitoneally on day 8 of the experiment. PBS: Phosphate-Buffered Saline.

Male ICR mice (20–25 g) were purchased from Hunan SJA Laboratory Animal Co. Ltd. (Changsha, China). Mice were kept in polycarbonate cages that were free of pathogens with wood chips for bedding at ambient temperature (22 ± 2 °C) in a 12-h light–dark cycle. Mice were separated into four groups after three days of acclimation (*n* = 10): (a) untreated mice (control group); (b) mice administered only LPS (LPS group); (c) mice administered LPS and berberine hydrochloride (50 mg/kg, berberine group) [27]; and (d) mice administered LPS and MCE (40 mg/kg, MCE group). The control and model groups received purified water by gavage daily. Other groups were given the same amount of berberine hydrochloride and MCE once daily for 8 days. Except for the control group, which was administered PBS intraperitoneally, the remaining groups were treated with LPS at 6 mg/kg intraperitoneally on day 8 of the experiment. On the final day of the research, mice were initially sedated and anesthetized using 10% chloral hydrate, intraperitoneally administered, and then euthanized by cervical dislocation (Figure 1). The European Community (Directive 2010/63/EU) criteria for laboratory animal care and use were rigorously followed in this work.

### 2.3. Diarrhea Index Determination

The diarrheagenic activity was assessed in the same way as previously described [28,29]. Mice were put in a box with a filter paper covering the bottom so that the fecal status could be observed. The healthy mice without spontaneous diarrhea were intraperitoneally injected with LPS (6 mg/kg). A positive diarrhea case was defined as any formless or liquid feces with a stain on the filter paper. In all experiments, there were 10 mice per group, and the observation period lasted 6 h. The diarrhea rate and diarrhea index are frequently employed as principal indicators in the standard methodology for developing a diarrhea model [30]. The diarrhea rate is determined by calculating the percentage of mice exhibiting diarrhea relative to the total number of mice. The diameters of the diluted/watery stools were graded as follows: 1 point for <1 cm, 2 points for 1–1.9 cm, 3 points for 2–3 cm, and 4 points for >3 cm. The diameter was measured for normal stools, and the average diameter of the irregular ones was determined by taking the mean of the longest and shortest diameters. Dilute stools were divided by total stools to establish each mouse’s dilute stool rate. The diarrhea index is calculated by multiplying the rate of dilute stool by its level.

### 2.4. Histological Study

Sections of the duodenum, jejunum, and ileum (*n* = 3 per group) were preserved in 4% paraformaldehyde. The fixed tissue underwent ethanol dehydration at varying concentrations, followed by paraffin embedding, sectioning, and hematoxylin and eosin (H&E) staining. The sections were examined using a NIKON DS-U3 photomicrography system (Nikon Corporation, Tokyo, Japan).

### 2.5. Serum IL-8 and TNF-α Assay

A glass capillary was used to obtain blood samples from the retroorbital venous plexus under anesthesia. The serum was centrifuged at 4000 rpm for 15 min. IL-8 and TNF-α were determined using an assay kit purchased separately from Elabscience Co., Ltd. (Wuhan, China) and Biopike Co., Ltd. (Beijing, China).

### 2.6. Analysis Using Quantitative Real-Time PCR

Total RNA was extracted from the duodenum using TRIzol reagent (Invitrogen, Carlsbad, CA, USA). The mRNA expression levels of *NF-κB*, *IL-6*, and *IL-1β* in the duodenum were quantified utilizing an Alytikjena qTOWER3G instrument (Analytik Jena, Jena, Germany). The specific primers employed in this analysis are detailed in Table 1. The purified RNAs were reversely transcribed using a SYBR^®^ Green Premix Pro Taq HS qPCR Kit (Accurate Biology, Changsha, China) according to the manufacturer’s instructions. Data normalization was performed using GAPDH as the reference gene.

### 2.7. Analysis of SCFAs in the Intestinal Substance

The intestinal substance was homogenized and centrifuged at 1000× *g* for 10 min at 4 °C. An amount of 4 mL of the supernatant was mixed with 1 mL of 25% metaphosphoric acid solution for the determination of SCFAs. Gas chromatography analysis was carried out as previously described [31].

### 2.8. 16S rRNA Gene Amplicon Sequencing

Twenty-four mice were randomly selected from the groups (6 per group) to undergo 16S rRNA sequencing analyses. Total genomic DNA was isolated from intestinal substance samples using QIAamp DNA Stool Mini Kits (QIAGEN, Hilden, Germany) as directed by the manufacturer. DNA concentration and purity were determined using a NanoDrop 2000 UV–vis spectrophotometer (Thermo Scientific, Wilmington, DE, USA) and 1% agarose gel electrophoresis.

The hypervariable region V3-V4 of the 16S rRNA gene was amplified with primer pairs 338F and 806R by using the thermocycler PCR system (ABI GeneAmp^®^ 9700, Applied Biosystems, Foster City, CA, USA). The 16S rRNA gene was amplified as previously described [32] and the PCR reactions were carried out in triplicate. The PCR product was purified and quantified using a QuantusTM Fluorometer (Promega, Madison, WI, USA). Finally, purified products were pooled and analyzed on an Illumina MiSeq PE300 system provided by Majorbio (Shanghai, China). The raw data were uploaded to the SRA database at the NCBI (BioProject ID: PRJNA914224).

### 2.9. Data Processing and Statistical Analysis

Data are presented as the mean ± standard error (SEM). Data normality and variance homoscedasticity were verified using Shapiro–Wilk and Levene’s tests, respectively, before executing the Tukey–Kramer post hoc test. A probability value of *p* < 0.05 was considered statistically significant, and *p* < 0.01 as highly significant. Bioinformatic analysis of the gut microbiota was carried out using the Majorbio Cloud platform (https://cloud.majorbio.com). Based on the OTU information, rarefaction curves and alpha diversity indices including observed OTUs, Chao1 richness, Shannon index and Good’s coverage were calculated with Mothur v1.30.1. The variation and similarity among the microbial communities in different samples were determined by partial least squares discriminant analysis (PLS-DA) using the mixOmics package (version 6.3.2). The PERMANOVA test was used to assess the percentage of variation explained by the treatment along with its statistical significance using the Vegan v2.5-3 package. The linear discriminant analysis (LDA) effect size (LEfSe) was performed to identify the significantly abundant taxa (phylum to genera) of bacteria among the different groups (LDA score > 2, *p* < 0.05). All standard statistical operations were performed using SPSS v25.0.

## 3. Results

### 3.1. MCE Alleviated LPS-Induced Diarrhea in Mice

**Figure 2:**
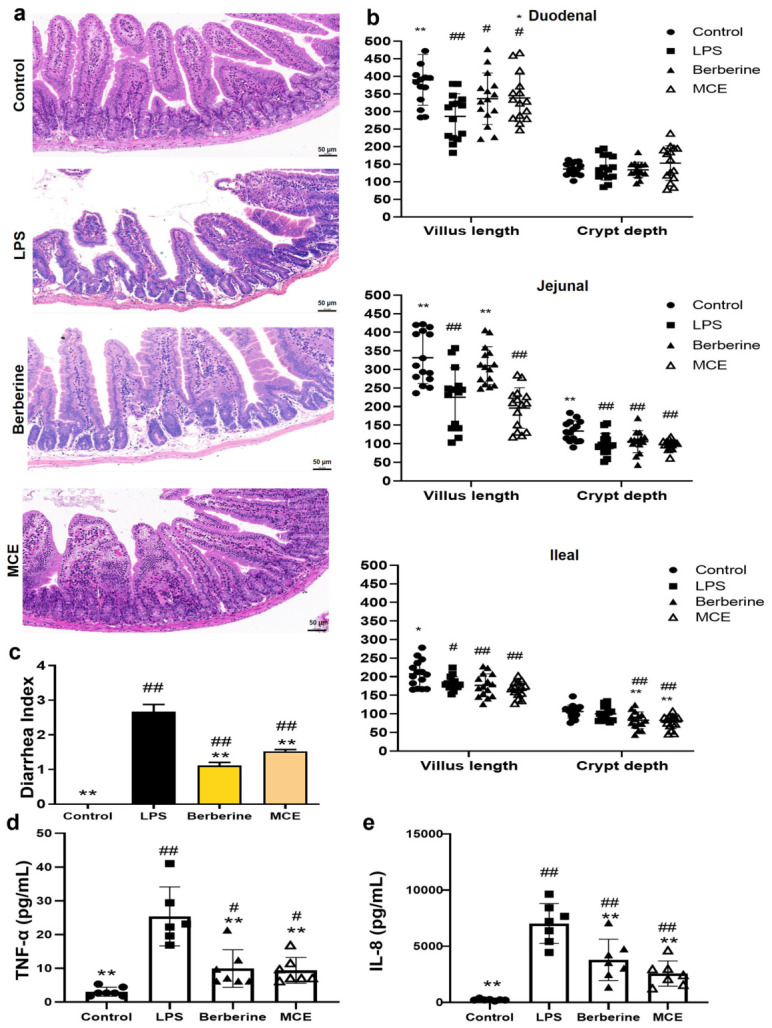
MCE alleviated LPS-induced diarrhea in mice. (**a**) Histological changes (H&E staining images of duodenal sections at original magnification; scale bar, 50 μm). (**b**) Villus length and crypt depth were measured for each group (*n* = 15). (**c**) Diarrhea index. (**d**) The levels of TNF-α (*n* = 6). (**e**) The levels of IL-8 (*n* = 6). Data are shown as mean ± SEM. # *p* < 0.05, ## *p* < 0.01, vs. the control group, * *p* < 0.05, ** *p* < 0.01 vs. the LPS group.

Half an hour after LPS injection, mice exhibited lethargy accompanied by ocular and nasal discharge and varying degrees of diarrhea, indicating successful induction of the diarrhea model. H&E staining revealed intact intestinal architecture in the control group, characterized by well-organized villi and normal crypt morphology. In contrast, LPS challenge resulted in pronounced intestinal damage, including villus shortening, crypt hyperplasia, goblet cell depletion, and inflammatory cell infiltration (Figure 2a and Figure S1). Treatment with MCE partially restored intestinal morphology, as evidenced by significantly increased villus height and reduced crypt depth compared with the LPS group (*p* < 0.01, Figure 2b). As shown in Figure 2c, the diarrhea index in the LPS group was significantly higher than that in the control group (*p* < 0.01). MCE administration markedly reduced the diarrhea index when compared to the LPS group (*p* < 0.01), and its antidiarrheal effect was comparable to that of berberine. These findings indicate that MCE effectively alleviates LPS-induced diarrhea and protects intestinal structural integrity.

To evaluate the systemic inflammatory response, serum levels of TNF-α and IL-8 were measured by ELISA. As depicted in Figure 2d,e, LPS administration significantly increased TNF-α and IL-8 concentrations (*p* < 0.01) in comparison to the CON group. Compared with the LPS group, MCE treatment dramatically reduced the LPS-induced increase in TNF-α and IL-8 levels, with reductions comparable to or greater than those observed in the berberine-treated group. These results indicated that MCE effectively mitigates systemic inflammation induced by LPS exposure.

### 3.2. Effects of MCE Treatment on Gut Microbiota Composition

16S rRNA gene sequencing was conducted to examine if the protective effects of MCE were linked to changes in gut microbiota. A total of 881, 831, 788, and 820 operational taxonomic units (OTUs) were identified in the control, LPS, berberine, and MCE groups, respectively (Figure S2). Among the four groups, 632 OTUs were shared, while 12 and 30 unique OTUs were detected in the berberine and MCE groups, respectively, indicating treatment-specific microbial alterations. Alpha diversity analysis demonstrated that the coverage values for all groups exceeded 0.99, suggesting enough sequencing depth (Figure S3). The absence of significant differences between groups suggests that the LPS challenge and subsequent treatments did not markedly affect overall microbial richness or diversity in the small intestine.

**Figure 3:**
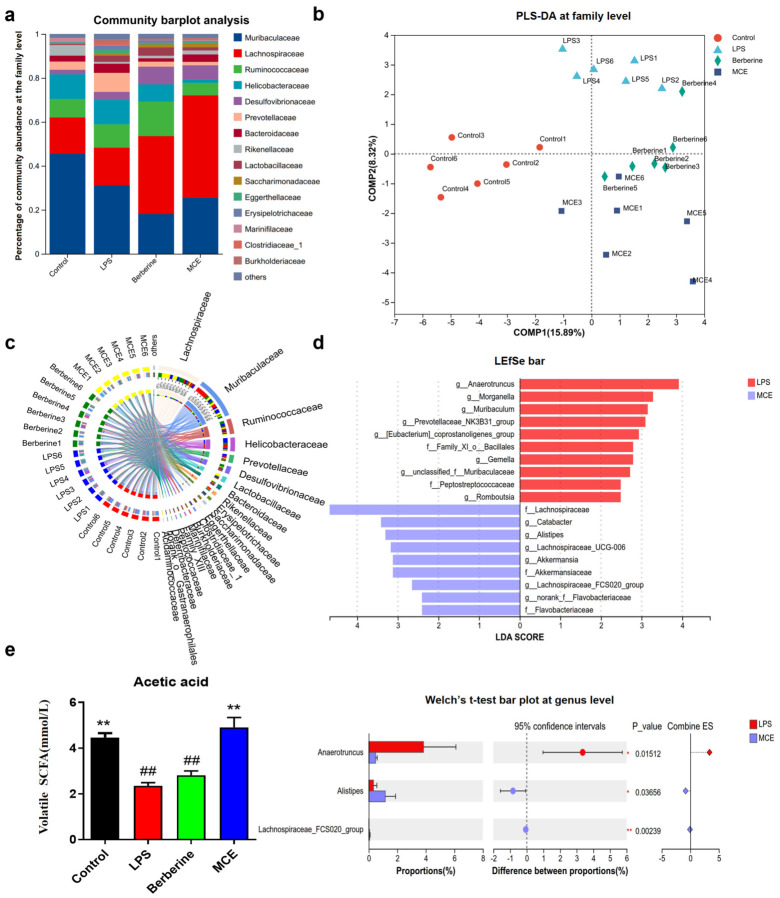
MCE altered the microbiota composition of mice. (**a**) Percentage of community abundance at the family level. (**b**) PLS-DA and (**c**) Circos at the genus level. (**d**) Test bar plot at the genus level and LEfSe bar between the MCE and LPS groups. (**e**) The concentration of acetic acid was determined by gas chromatography. PLS-DA, partial least squares discriminant analysis; LEfSe, linear discriminant analysis effect size. Data are shown as mean ± SEM. ## *p* < 0.01, vs. the control group, * *p* < 0.05, ** *p* < 0.01 vs. the LPS group.

At the family level, notable differences in microbial composition were observed between groups (Figure 3a). *Muribaculaceae* was the dominant family in the control group (45.56%) but decreased markedly in the LPS group (31.05%) and further in the berberine group (18.18%). In contrast, MCE treatment partially restored *Muribaculaceae* abundance (25.59%). *Lachnospiraceae* accounted for 16.50% of the microbial community in the control group but increased substantially in the LPS (17.21%), berberine (35.42%), and MCE groups (46.52%). *Ruminococcaceae* abundance was highest in the berberine group (15.68%) but decreased in the MCE group (5.67%). *Helicobacteraceae* showed the most pronounced reduction in the MCE group (1.48%). The PLS-DA score plots revealed a distinct separation between the MCE group and LPS group, suggesting the overall structures of the bacterial communities in the groups were significantly different (Figure 3b). Circos analysis further revealed that *Lachnospiraceae* and *Muribaculaceae* constituted the predominant shared taxa among the LPS, berberine, and MCE groups (Figure 3c).

The diverse taxa and their dominant bacteria in the intestinal microflora of mice under the intervention of different drugs are shown in Figure 3d. A total of 20 taxonomic biomarkers were identified by LEfSe analysis based on an LDA score > 2.0. Specifically, *Anaerotruncus*, *Morganella*, and *Muribaculum* were significantly enriched in the red group, with *Anaerotruncus* acting as the primary contributor. Conversely, the blue group was characterized by a predominant enrichment of the *Lachnospiraceae* family, alongside the genera *Catabacter*, *Alistipes*, and *Akkermansia*. To further confirm these alterations, Welch’s t-test was performed at the genus level. The relative abundance of *Anaerotruncus* was significantly increased in the LPS group compared with the MCE group (*p* = 0.015). In contrast, the blue group exhibited a higher proportion of Alistipes (*p* = 0.031) and a highly significant enrichment of the Lachnospiraceae_FCS020_group (*p* = 0.002). Intriguingly, when cross-compared with the berberine group, the MCE group still exhibited a distinct and robust enrichment of specific microbes (Figure S4). Both LEfSe and Welch’s t-test confirmed that *g__norank_f__Eggerthellaceae* and *g__Tyzzerella* were highly significantly enriched in the MCE group compared to the berberine group. These findings indicate that while both are natural alkaloids, MCE possesses a unique pharmacological niche in recruitment of functional microbes like *Lachnospiraceae* and *Eggerthellaceae*, distinguishing its therapeutic pathway from other conventional compounds.

The analysis of SCFAs demonstrated a significant reduction in acetic acid content in both the LPS and berberine treatment groups compared to the control group (Figure 3e; Table S1). Notably, MCE treatment restored acetic acid levels to values comparable to those of the control group. In contrast, other SCFAs showed no consistent or significant changes among groups.

**Figure 4:**
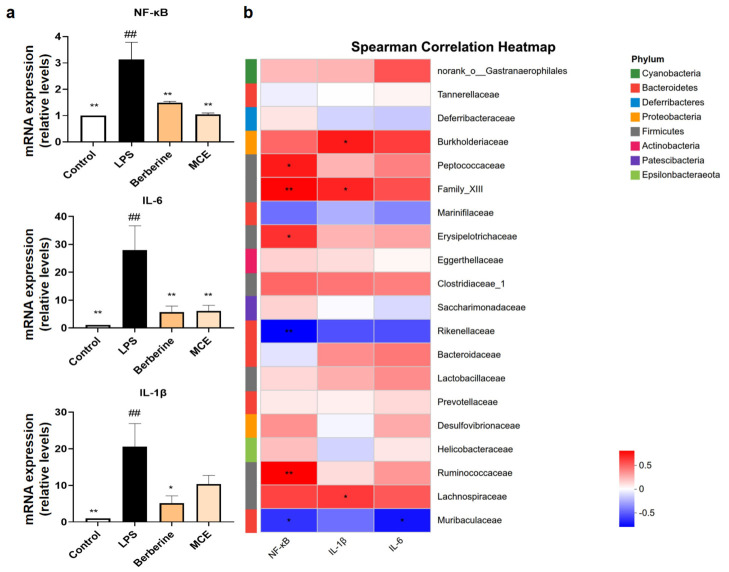
MCE improves intestinal inflammatory factors and related intestinal flora. (**a**) *IL-1β*, *IL-6* and *NF-κB* mRNA in the duodenum. (**b**) Spearman correlation analysis between *IL-1β*, *IL-6* and *NF-κB* and intestinal flora. *IL*, interleukin; *TNF-α*, tumor necrosis factor-α; *NF-κB*, Nuclear Factor Kappa B. Data are shown as mean ± SEM. ## *p* < 0.01, vs. the control group, * *p* < 0.05, ** *p* < 0.01 vs. the LPS group.

To further explore the relationship between gut microbiota and inflammation, mRNA expression levels of *IL-1β*, *IL-6*, and *NF-κB* in the duodenum were quantified. As shown in Figure 4a, in contrast to the control group, LPS notably upregulated the mRNA expression of *IL-1β*, *IL-6*, and *NF-κB*, whereas both berberine and MCE treatments markedly attenuated their expression. The expression of *IL-1β* was significantly and positively linked to *Burkholderiaceae* and *Lachnospiraceae*, while *Ruminococcaceae* and *Family_XIII* exhibited strong positive correlations with *NF-κB*. Conversely, a significant negative Spearman correlation was identified for *Muribaculaceae* (against *NF-κB* and *IL-6*) and *Rikenellaceae* (against *NF-κB*) (Figure 4b).

## 4. Discussion

Diarrhea induced by lipopolysaccharide is characterized by acute intestinal inflammation, epithelial barrier disruption, and impaired fluid and nutrient absorption [33]. This study shows that *Macleaya cordata* extract effectively mitigates LPS-induced diarrhea in mice. This protective effect was accompanied by improvements in intestinal morphology, suppression of systemic and local inflammatory responses, modulation of gut microbiota composition, and restoration of acetic acid levels. Collectively, these findings indicate that MCE exerts its antidiarrheal activity through coordinated regulation of the gut microbiota–metabolite–inflammation axis.

Anti-inflammatory, immunomodulatory and antioxidant activities are vital for host health [34]. Cytokines are bioactive molecules secreted by activated immune cells, which mediate cellular crosstalk and play key roles in immune responses, inflammation, and tissue hematopoiesis and repair [35,36,37]. Excessive activation of inflammatory signaling pathways is a central mechanism underlying LPS-induced intestinal injury [38]. LPS activates innate immunity via Toll-like receptor 4, triggering NF-κB and the transcription of pro-inflammatory cytokines such as TNF-α, IL-1β, and IL-6 [39,40]. Sustained elevation of these mediators disrupts epithelial tight junctions, increases intestinal permeability, and exacerbates diarrheal symptoms [41]. MCE markedly reduced serum TNF-α and IL-8 levels and significantly suppressed the intestinal mRNA expression of *IL-1β*, *IL-6*, and *NF-κB*. These results are consistent with previous reports demonstrating the anti-inflammatory properties of MCE. Thus, inhibition of NF-κB-mediated inflammatory signaling appears to be a key mechanism by which MCE protects against LPS-induced intestinal injury.

Intestinal morphology provides a direct indication of epithelial integrity and absorptive capacity [42,43,44]. Villus shortening and crypt hyperplasia are commonly associated with inflammatory diarrhea and reflect impaired epithelial renewal and nutrient absorption [45,46]. In this study, LPS challenge resulted in marked villus atrophy and crypt deepening in the duodenum, whereas MCE treatment significantly ameliorated these structural abnormalities. Preservation of villus height and normalization of crypt depth suggest that MCE helps maintain epithelial homeostasis under inflammatory stress, thereby contributing to improved intestinal function and reduced diarrhea severity.

Gut microbiota research in animals and humans has advanced, revealing its multifaceted functions ranging from alleviating neurological disorders through metabolic pathways [47] to bolstering host intestinal health and immune systems [48]. Increasing evidence underscores the pivotal function of intestinal microflora in modulating intestinal inflammation and barrier integrity [49,50]. Rather than inducing broad changes in microbial diversity, MCE selectively altered the relative abundance of specific bacterial taxa. Analysis indicates that MCE treatment distinctively shapes the gut microbiota, promoting beneficial bacteria associated with anti-inflammatory effects. Specifically, MCE administration drove a significant increase in the family *Lachnospiraceae*, a major group of SCFA producers that strengthen the intestinal barrier, which aligns with findings regarding enhanced tissue integrity and reduced diarrhea [51,52]. Furthermore, compared to the berberine group, the MCE group showed a unique enrichment in *g__norank_f__Eggerthellaceae*. This family is known to be involved in the metabolic transformation of natural compounds in the gut [53], suggesting a distinct mechanism for MCE. These findings support a specific, beneficial microbial modulation pattern for MCE. 

SCFAs are key microbial metabolites that mediate host–microbiota interactions and play essential roles in intestinal homeostasis [54]. Among SCFAs, acetate is particularly important for promoting sodium and water absorption, enhancing epithelial barrier integrity, and modulating immune responses [55,56,57]. Acetic acid can modulate intestinal immune responses by activating G protein-coupled receptors, which inhibits the release of pro-inflammatory cytokines and helps to alleviate intestinal inflammation [58]. In the present study, acetic acid levels were significantly reduced following LPS challenge and were restored by MCE treatment to levels comparable with those of the control group. Interestingly, this effect differed from that of berberine, which has been reported to preferentially enhance butyrate production [59,60]. This divergence indicates that MCE and berberine exert antidiarrheal effects via compound-specific modulation of microbial metabolic pathways; MCE restores acetic acid production while berberine preferentially enhances butyrate synthesis, which provides a theoretical basis for their selective application in clinical practice.

## 5. Conclusions

In summary, our results confirmed that MCE effectively alleviated LPS-induced diarrhea in mice by attenuating intestinal and systemic inflammatory responses and preserving intestinal epithelial structure. These findings suggest that the protective effects of MCE are closely associated with the modulation of gut microbiota and the enhancement of acetic acid production, although further functional validation is required to establish direct causality. While these mechanisms were identified in a murine model, they establish a solid theoretical baseline to inform future in vivo trials in livestock. Crucially, this study highlights the potential of MCE in preventing or treating bacterial diseases linked to LPS pathogenesis, offering a compelling mechanistic basis for developing MCE as a natural antibiotic alternative to prevent inflammatory diarrhea in animal husbandry.

## Figures and Tables

**Table 1:** Sequences of primers.

Gene	Primer Sequence (5′-3′)	Accession Number
*GAPDH*	CCTCGTCCCGTAGACAAAATG TGAGGTCAATGAAGGGGTCGT	NM_008084.2
*NF-κB*	TCCTTTTCTCAAGCTGATGTGC TTTCGGGTAGGCACAGCAAT	NM_001365067.1
*IL-6*	AGACTTCCATCCAGTTGCCT CAGGTCTGTTGGGAGTGGTA	NM_001314054.1
*IL-1β*	ACTCATTGTGGCTGTGGAGA TTGTTCATCTCGGAGCCTGT	NM_008361.4

Abbreviations: *GAPDH*, glyceraldehyde 3-phosphate dehydrogenase; *IL*, interleukin; *TNF-α*, tumor necrosis factor-α; *NF-κB*, Nuclear Factor Kappa B.

## Data Availability

The data used to support the results of the present study can be obtained from the corresponding authors.

## Supplementary Materials

The following supporting information can be downloaded at https://www.mdpi.com/article/10.3390/ani16121922/s1, Figure S1. H&E staining of small intestin at original magnificatione, (a) Duodenum, (b) Jejunum, (c) Ileum. Scale bar, 50 μm. Figure S2. Number of bacterial OTUs in LPS-induced mice feces. The (a) OTU level and (b) sparse curve in LPS-induced mice feces. Figure S3. α-diversity analysis of the intestinal microbiota in LPS-induced mice. The detection of (a) Chao, (b) Shannon, (c) Ace, (d) Simpson, (e) sobs, and (f) coverage index in LPS-induced mice feces (*n* = 6 per group). Statistical significance and pairwise comparisons were determined by One-way analysis of variance (ANOVA) followed by the Tukey-Kramer post-hoc test. Figure S4. LEfSe and Welch’s t-test analyses of the gut microbiota between the MCE and Berberine groups. (a) LEfSe bar plot. (b) Welch’s t-test bar plot. Data are presented as the mean ± SEM (*n* = 6). (* *p* < 0.05) and (** *p* < 0.01). Table S1. VFA of *Macleaya cordata* extract and berberine treatment of mouse (mmol/L).
