# Supplementary material for: Effects of Macleaya Cordata Extract on LPS-Induced Intestinal Inflammation and Diarrhea via Modulation of Gut Microbiota
Source: Animals (Basel). 2026 Jun 22;16(12):1922. doi: 10.3390/ani16121922 (PMC13296292; doi:10.3390/ani16121922)
Supplement: Supplementary file 1 [file animals-16-01922-s001.zip › animals-4334466-supplementary.pdf]

Supplementary Material

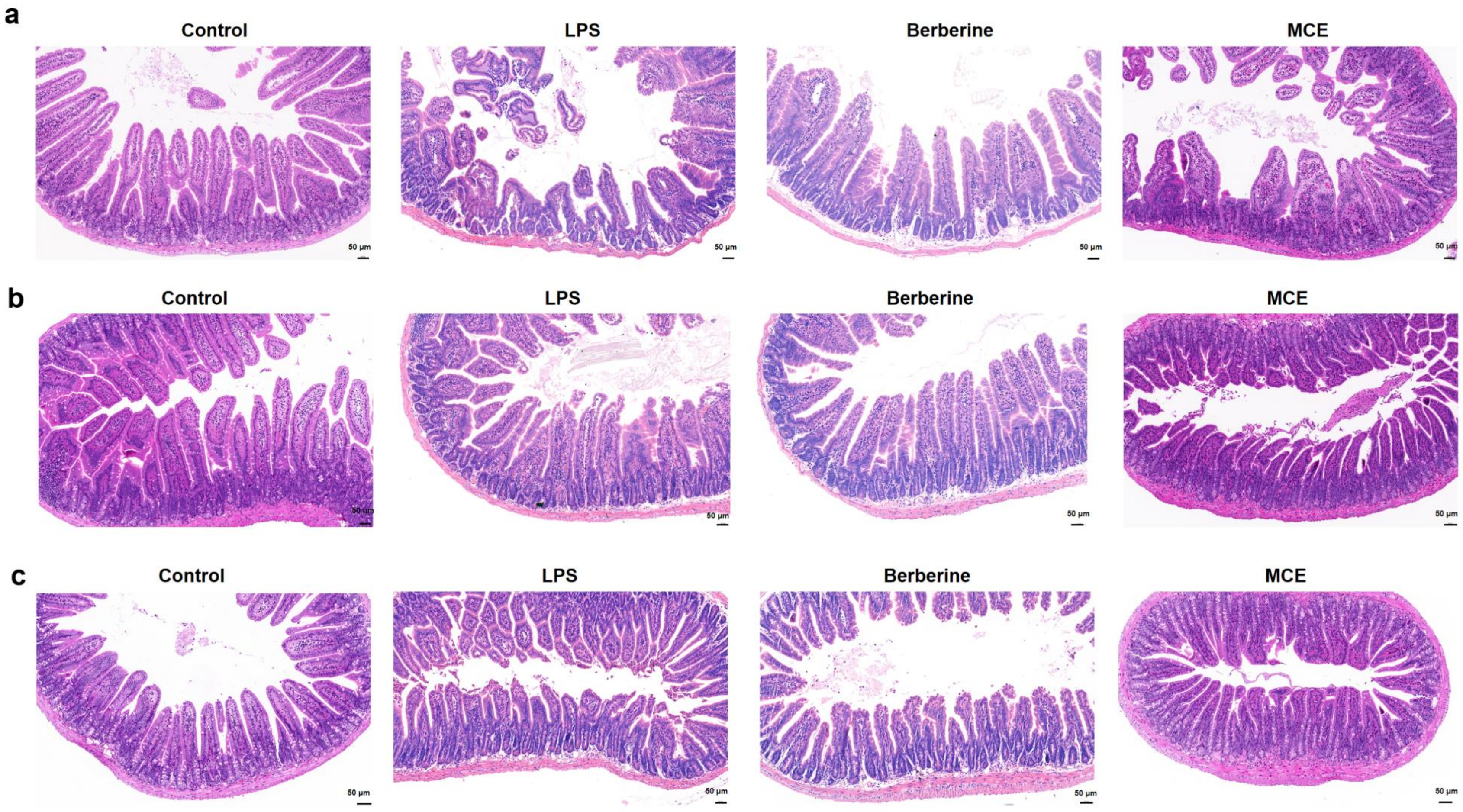

Figure S1. H&E staining of small intestin at original magnificatione, (a) Duodenum, (b) Jejunum, (c) Ileum. Scale bar, 50 µm.

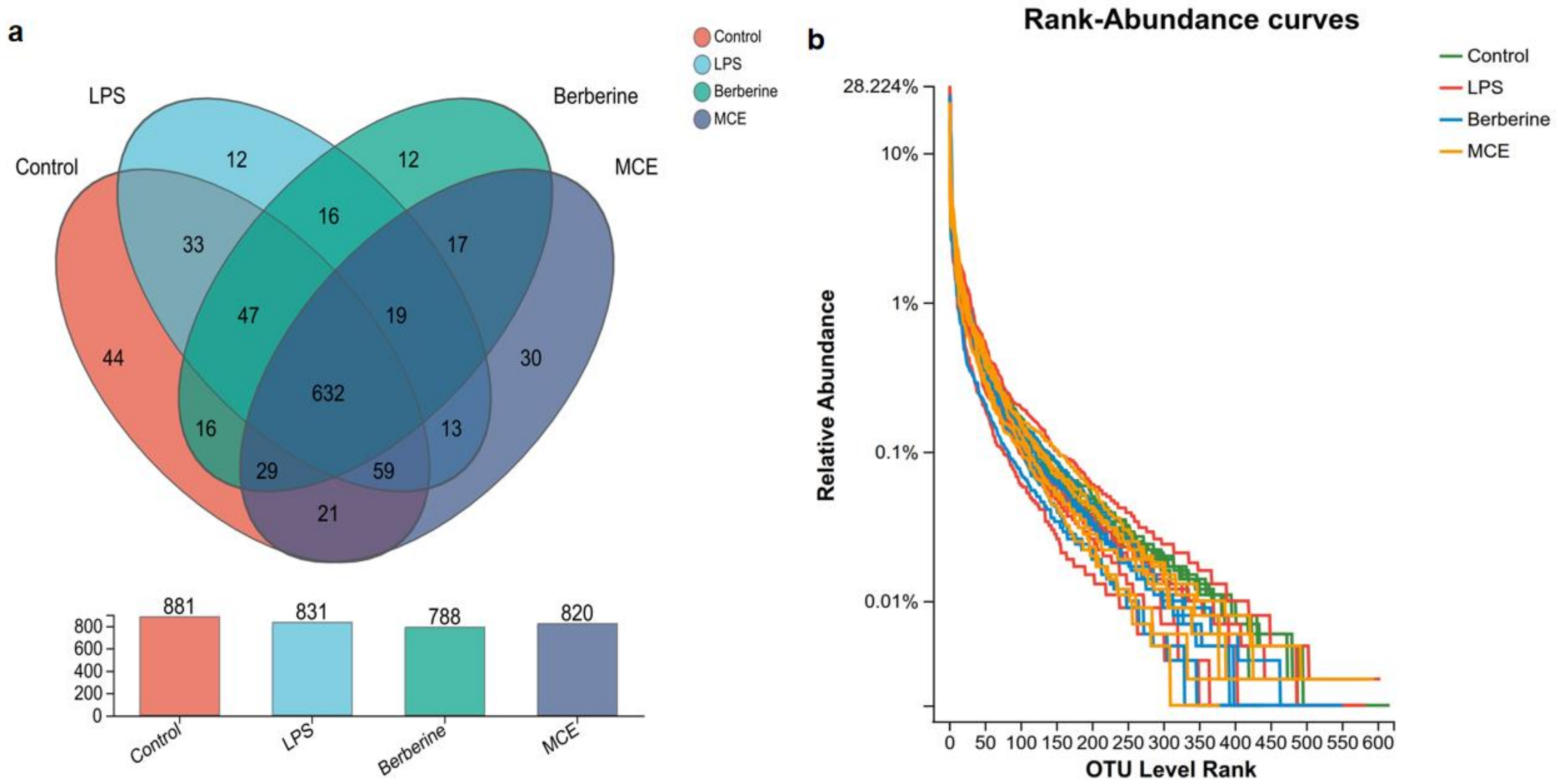

Figure S2. Number of bacterial OTUs in LPS-induced mice feces. The (a) OTU level and (b) sparse curve in LPS-induced mice feces.

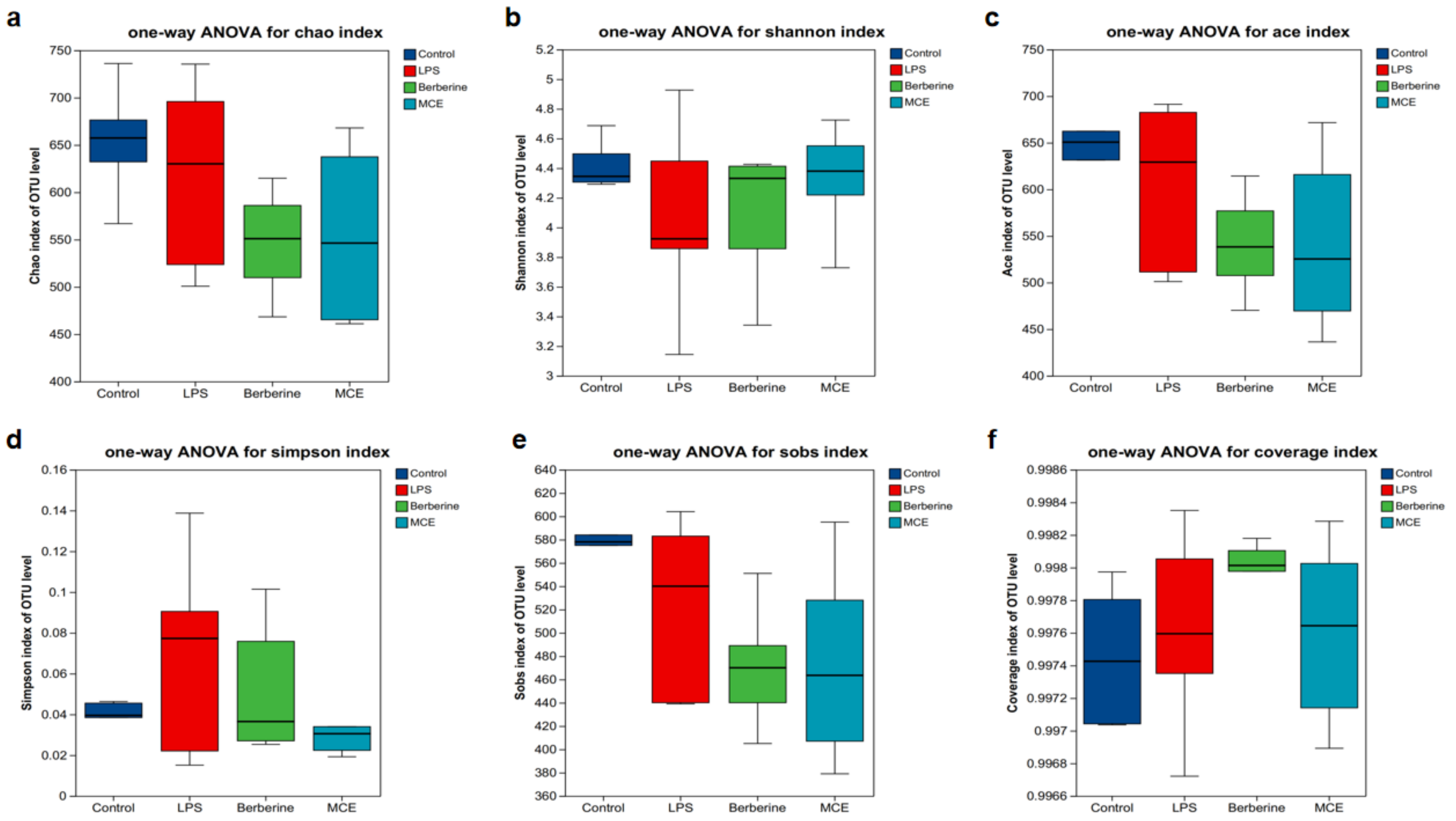

Figure S3. α-diversity analysis of the intestinal microbiota in LPS-induced mice. The detection of (a) Chao, (b) Shannon, (c) Ace, (d) Simpson, (e) sobs, and (f) coverage index in LPS-induced mice feces (n=6 per group). Statistical significance and pairwise comparisons were determined by One-way analysis of variance (ANOVA) followed by the Tukey-Kramer post-hoc test.

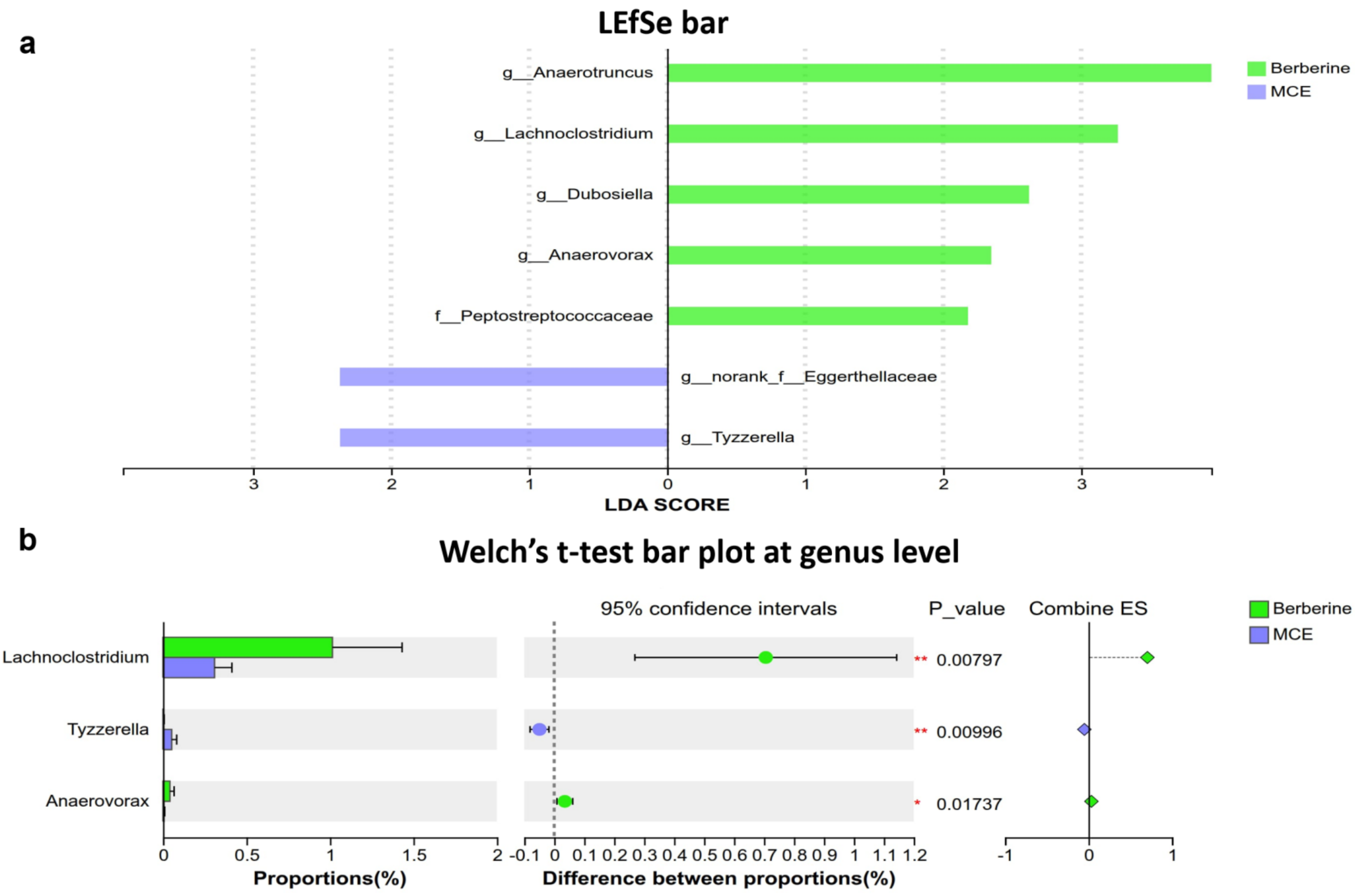

Figure S4. LEfSe and Welch's t-test analyses of the gut microbiota between the MCE and Berberine groups.(a) LEfSe bar plot. (b) Welch's t-test bar plot. Data are presented as the mean ± SEM (n = 6). (*$P < 0.05$) and (**$P < 0.01$).

Table S1

VFA of *Macleaya cordata* extract and berberine treatment of mouse (mmol/L).

	Control	LPS	Berberine	MCE
Acetic acid	4.46 ± 0.189^{a}	2.35 ±0.139^{b}	2.81 ± 0.139^{b}	4.90 ± 0.434^{a}
Propionic acid	0.92 ± 0.062^{a}	0.52 ± 0.018^{b}	0.70 ± 0.022^{c}	0.67 ± 0.095^{c}
Isobutyric acid	0.07 ± 0.005^{a}	0.06 ± 0.003^{a}	0.11 ± 0.006^{b}	0.09 ± 0.013^{ab}
Butyric acid	0.58 ± 0.040^{a}	0.42 ± 0.019^{b}	0.58 ± 0.023^{a}	0.59 ± 0.074^{a}
Isovaleric acid	0.08 ± 0.006^{a}	0.09 ± 0.007^{a}	0.13 ± 0.004^{b}	0.13 ± 0.023^{b}
Valeric acid	0.04 ± 0.003^{ab}	0.03 ± 0.003^{a}	0.07 ± 0.005^{c}	0.05 ± 0.005^{b}
Volatile fatty acid	6.16 ± 0.257^{a}	3.46 ± 0.169^{b}	4.40 ± 0.216^{b}	6.44 ± 0.625^{a}

Data are presented as the mean ± standard error of the mean (SEM) (n = 10). Within the same row, values with different superscript letters (a, b, c) indicate a significant difference ($P < 0.05$), as determined by One-way ANOVA.
